# Data-independent acquisition proteomic analysis of the brain microvasculature in Alzheimer’s disease identifies major pathways of dysfunction and upregulation of cytoprotective responses

**DOI:** 10.1186/s12987-024-00581-1

**Published:** 2024-10-21

**Authors:** Michelle A. Erickson, Richard S. Johnson, Mamatha Damodarasamy, Michael J. MacCoss, C. Dirk Keene, William A. Banks, May J. Reed

**Affiliations:** 1 Department of Medicine, Division of Gerontology and Geriatric Medicine, University of Washington, Harborview Medical Center, 325 9th Avenue, Seattle, WA 98104 USA; 2 Geriatric Research Education and Clinical Center, VA Puget Sound Health Care System, 1660 S. Columbian Way, Seattle, WA 98108 USA; 3 Department of Genome Sciences, University of Washington, Seattle, USA; 4 Department of Laboratory Medicine and Pathology, Division of Neuropathology, University of Washington, Seattle, WA USA

**Keywords:** Alzheimer’s disease, Blood–brain barrier, Brain microvessels, Neurovascular unit, Proteomics

## Abstract

Brain microvascular dysfunction is an important feature of Alzheimer’s disease (AD). To better understand the brain microvascular molecular signatures of AD, we processed and analyzed isolated human brain microvessels by data-independent acquisition liquid chromatography with tandem mass spectrometry (DIA LC–MS/MS) to generate a quantitative dataset at the peptide and protein level. Brain microvessels were isolated from parietal cortex grey matter using protocols that preserve viability for downstream functional studies. Our cohort included 23 subjects with clinical and neuropathologic concordance for Alzheimer’s disease, and 21 age-matched controls. In our analysis, we identified 168 proteins whose abundance was significantly increased, and no proteins that were significantly decreased in AD. The most highly increased proteins included amyloid beta, tau, midkine, SPARC related modular calcium binding 1 (SMOC1), and fatty acid binding protein 7 (FABP7). Additionally, Gene Ontology (GO) enrichment analysis identified the enrichment of increased proteins involved in cellular detoxification and antioxidative responses. A systematic evaluation of protein functions using the UniProt database identified groupings into common functional themes including the regulation of cellular proliferation, cellular differentiation and survival, inflammation, extracellular matrix, cell stress responses, metabolism, coagulation and heme breakdown, protein degradation, cytoskeleton, subcellular trafficking, cell motility, and cell signaling. This suggests that AD brain microvessels exist in a stressed state of increased energy demand, and mount a compensatory response to ongoing oxidative and cellular damage that is associated with AD. We also used public RNAseq databases to identify cell-type enriched genes that were detected at the protein level and found no changes in abundance of these proteins between control and AD groups, indicating that changes in cellular composition of the isolated microvessels were minimal between AD and no-AD groups. Using public data, we additionally found that under half of the proteins that were significantly increased in AD microvessels had concordant changes in brain microvascular mRNA, implying substantial discordance between gene and protein levels. Together, our results offer novel insights into the molecular underpinnings of brain microvascular dysfunction in AD.

**Supplementary Information:**

The online version contains supplementary material available at 10.1186/s12987-024-00581-1.

## Background

Alzheimer's disease (AD) is a progressive and deadly neurodegenerative disease for which there is not a cure. Multiple factors, such as brain microvascular dysfunction, contribute to AD pathophysiology. Consequences of brain microvascular dysfunction include impaired cerebral blood flow regulation, breakdown of the extracellular matrix leading to vascular fragility and degeneration, and altered functions of the blood–brain barrier (BBB) [1–4]. The BBB is primarily comprised of specialized endothelial cells that are simultaneously exposed to both the brain and blood compartments. Brain endothelial cells are specialized in that they largely prevent the unregulated leakage of substances between the brain and blood compartments across either paracellular or transcellular routes. In addition to their important barrier functions, brain endothelial cells also express transporters that selectively regulate the passage of vital substances into and out of the brain. These important and unique functions of brain endothelial cells are conferred, in part, by closely apposed pericytes and astrocytic endfeet, as well as other brain cell types that interact with the brain microvasculature and regulate its functions [5]. Dysfunctions of the BBB that have been associated with AD include BBB disruption [6], defined here as the unregulated leakage of circulating substances into the brain, impaired clearance and subsequent buildup of toxic proteins in the brain such as amyloid beta protein [7, 8], impaired or exacerbated transport of substances into the brain [9–11], altered trafficking of immune cells [12, 13], and changes in secretions of brain endothelial cells [14]. Each of these aspects of BBB and brain microvascular dysfunction likely contribute to AD etiology and progression. However, the molecular underpinnings of brain microvascular dysfunction in AD are incompletely understood.

Omics-based studies to date have greatly improved our understanding of the vast molecular changes in brain tissues and brain cell types that occur in AD, particularly those that have been done using human brain tissues. For example, single-cell and single-nucleus RNAseq methods have identified important cell-type specific changes in the transcriptome that occur in the brain with AD [15]. It has also recently been shown in bulk brain tissue that there are proteomic changes with AD that are not detected at the RNA level, particularly those affecting the extracellular matrix and aspects of signaling and metabolism [16]. To date, few studies have evaluated global proteomic profiles of the brain microvasculature in human postmortem AD brain tissues [17–19]. Here, we present a quantitative dataset at the peptide and protein level of microvessels isolated from parietal cortex grey matter obtained from rapid autopsies of 21 control and 23 subjects with AD. Samples are well-matched for age within sex, post-mortem interval, and have concordant clinical and neuropathological diagnoses. The isolation techniques used here are optimized to obtain highly pure brain microvessel isolates that retain their viability and functionality in culture [20]. We evaluate the overall changes in proteins detected with AD, and concordance of changes detected in the full data set among male and female donor groups. We determine the enriched molecular pathways reflected in AD brain microvasculature using GO analysis, as well as functions that are shared among proteins found to be increased in AD. Using publicly available data and databases, we assess overlapping and uniquely increased proteins in isolated brain MVs vs. bulk brain tissue, the presence and abundance of cell-type specific protein markers of the neurovascular unit, and the extent to which there is altered expression at the mRNA level of proteins identified to be significantly increased in AD. Our findings offer insight into biological processes and novel molecular targets that become altered in the brain microvasculature in AD.

## Methods

### Isolation of human brain microvessels from rapid autopsies

All tissues were derived from brains donated for research from participants in the University of Washington (UW) Neuropathology Core, which includes the UW Alzheimer’s Disease Research Center (ADRC) and the Kaiser Permanente Washington Health Research Institute (KPWHRI) Adult Changes in Thought (ACT) study with informed consent under protocols approved by the Institutional Review Board (IRB) at UW and KPWHRI. The UW Human Subjects Division deems the use of pre-existing de-identified samples as non-human subjects' research. Human brain samples were collected on a continuous basis from the UW BioRepository and Neuropathology (BRaIN) laboratory and Precision Neuropathology Core, which performs rapid autopsies (postmortem interval approximately 12 h or less) [21, 22]. For this project, upon brain removal in a rapid autopsy, a portion of the superior parietal lobule was dissected and cryostored at -80°C or processed immediately after dissection without freezing. The superior parietal lobe was selected as a region that demonstrates AD-associated neuropathologic [23] and metabolic changes and is consistently obtained from rapid autopsies in quantities that are optimal for microvessel isolations. Brain sections were placed in 4 °C endothelial cell (EC) media (Science Cell Research Laboratories, Carlsbad, CA – Catalog #1001) with 5% FBS (Science Cell Research Laboratories, Carlsbad, CA FBS, Cat. #0025); frozen brains were thawed in media with 5% FBS prior to further processing. The distribution of frozen vs. fresh brain tissue was equal in each group and shown in Table S1. Subsequently, human microvessels (MV)s were isolated from the deidentified parietal cortex as previously described [20]. Briefly, cerebral cortex was dissected away from subcortical white matter in cold media with 5% FBS on ice and homogenized in a Dounce type homogenizer and centrifuged at 2000g for 5 min at 4°C. Supernatant was subsequently drawn off and clean absorbent pads used to remove residual supernatant/media. The pellet was resuspended in 10 ml of 15% (w/v) dextran and centrifuged at 10,000g for 20 min at 4°C. The pellet containing enriched brain microvessels was resuspended in 1 ml Dulbecco’s Phosphate Buffered Saline (DPBS), transferred to a 40 µm cell strainer (Thermo Fisher Scientific, Waltham, MA, Cat. #352340) and washed with 10ml of cold DPBS to remove single cells such as red blood cells. Finally, the strainer was reversed and microvessels retrieved using 2–3 ml of media or DPBS with 0.5% (w/v) BSA, then rinsed with DPBS, and centrifuged at 2000g for 3 min at 4 °C. MV pellets were washed three times in sterile DPBS to remove BSA prior to utilizing. All microvessels were assessed via light microscopy under 100 × magnification to confirm the purity of each microvessel isolation. Additionally, we verified the purity of a representative microvessel isolation by western blotting for the neuronal marker microtubule associated protein (MAP2) and the endothelial cell marker claudin-5 in the final microvascular pellet and the capillary depleted brain fraction recovered from the top of the dextran gradient (Figure S1).

With each de-identified sample, relevant demographic and study data including age, sex, race, and clinical dementia diagnosis are available; *APOE* genotype is also available in all but one donor. In subsequent analyses, neuropathological data from the donor brain including brain weight, AD neuropathologic change (ADNC) assessments, other neurodegenerative processes, and vascular brain injury are established. We utilized ADNC to select and stratify donors for this study; ADNC incorporates a measure of Aβ plaque distribution across the brain (Thal phase), neurofibrillary (pTau) tangle distribution across the brain (Braak stage), and cerebral cortical neuritic plaque density (CERAD score) [23]. For the series utilized herein, we were able to obtain n = 28 female and n = 16 male subjects with concordant clinical and neuropathological diagnosis of AD (cognitive status dementia, Braak V, VI and Overall ADNC high: 15 female, 8 male) or controls with no AD (cognitive status no dementia, Braak 0-IV and Overall ADNC not or low: 13 female, 8 male) who were age-matched within sex. Post-mortem intervals were 12 h or less. Table 1 summarizes the demographics of donors used in this current study and Table S1 shows the demographics for each individual donor.

**Table 1:** Subject demographics summary

	Females		Males		Females and males	
	No Dementia	Dementia	No dementia	Dementia	No dementia	Dementia
Number of Subjects	13	15	8	8	21	23
Age at Death (± SD)	91.54 ± 6.118	91.67 ± 6.218	78.25 ± 13.75	74.75 ± 10.22	86.48 ± 11.50	85.78 ± 11.21
Post-mortem Interval (± SD)	5.969 ± 1.499	6.367 ± 1.79	8.325 ± 2.598	5.913 ± 1.541*	6.867 ± 2.255	6.209 ± 1.686
*APOE4*%	0	20	0	50	0	30.4
Fresh %	30.1	33.3	37.5	37.5	33.3	34.8

*p < 0.05 vs. male no dementia group

### Proteomic analysis

#### Microvessel tryptic digestion

To each microvessel sample (wet pellet of 10–15 µl) was added 12 µl of 2 × lysis buffer (4 M urea, 10% SDS, 200 mM triethylammonium bicarbonate [TEAB], 20 mM tris (2-carboxyethyl) phosphine hydrochloride [TCEP], 2% HALT protease inhibitor (Thermo Scientific), and 24 ng/µl yeast enolase as a digestion control. The samples were bath sonicated on ice for 15 min, and then vortexed at 1400 rpm at 37 °C for one hour to reduce disulfide bonds and solubilize the samples. The samples were cooled to room temperature, and the free thiols were alkylated for 30 min after the addition of 2 µl of 500 mM iodoacetamide. Digestion and sample cleanup was performed using micro-S-traps (Protifi) as follows. Each sample was acidified by the addition of 2.5 µl 27.5% phosphoric acid, and then protein was precipitated by the addition of 165 µl binding buffer (90% methanol, 10% 1 M TEAB). The resulting precipitation was loaded onto a micro-S-trap via centrifugation at 4000g. The precipitated protein was then washed once with 150 µl of binding buffer, 150 µl of 1:1 chloroform/methanol, and then three more times with 150 µl binding buffer. After the final wash of binding buffer, the samples were centrifuged one more time to remove all binding buffer. A 20 µl aliquot of 50 mM TEAB containing 2 µg porcine trypsin (sequencing grade, Thermo Scientific) was added to the captured and washed protein precipitate, and then incubated for two hours at 47 °C. The resulting tryptic peptides were eluted from the trap by washing out with 40 µl 50 mM TEAB, 40 µl 0.1% formic acid, and finally with 40 µl of 50% acetonitrile and 0.1% formic acid in water. The combined eluates were dried on a vacuum centrifuge and resolubilized in 50 µl 0.1% trifluoroacetic acid (TFA) in water that contained 10 fmol/µl peptide retention time calibration (PRTC) standard (Thermo Scientific). The sample preparations and data acquisition were done in three batches, and for each batch a pooled sample was created by combining a portion of each sample within each batch.

Liquid Chromatography/Mass Spectrometry (LC/MS): All mass spectrometry was performed on an Exploris 480 (Thermo Fisher Scientific) mass spectrometer with a Thermo Easy-nLC HPLC with autosampler. Samples were injected via autosampler at volumes of 3 µl each onto a 150-μm Kasil fritted trap (Dr. Maisch Reprosil-Pur 120 C18-AQ 3 µm beads, 2 cm × 150 µm) at a 2 µl/min flow rate. The trap was desalted with 8ul of loading buffer and then brought on-line with a PepSep (Bruker) 150 µm × 150 cm column packed with 1.9 µm C18 beads. A zero dead volume connector was used to attach the column outlet to a Fossiliontech 20 µm ID emitter. The trap and column were mounted to a nanospray ion source (CorSolutions, Ithaca, NY) heated to 45ºC, and placed in line with the HPLC pump. A gradient of 2–32% acetonitrile in 0.1% formic acid was used to elute peptides off the column over 90 min. Operation of the mass spectrometer used electrospray ionization (2.5 kV) with the heated transfer tube at 300°C using methods of data independent acquisition (DIA).

For DIA of individual samples, one MS1 spectrum (m/z 395-1005, 30,000 resolution) was acquired with every 75 targeted MS2 spectra, with the targeted m/z value set to m/z 404.4337. The m/z was then sequentially increased by 8.0036 up to 1000.704. After another MS1 scan, a new cycle of targeted MS2 scans was initiated where the center of each isolation window was staggered by m/z 4.0018 compared to the first round of MS2 (in other words, starting at m/z 400.4319, stepping up by m/z 8.0036 for each MS2, and ending with m/z 996.703). This data acquisition pattern was repeated throughout each run (MS1, followed by 75 targeted MS2, followed by MS1, followed by targeted MS2 offset by m/z 4.0018). An effective precursor isolation width of m/z 4.0018 can be achieved by deconvolving this staggered precursor range [24]. The use of non-integer targeted MS2 precursor values (ie, m/z 404.4337 instead of m/z 404) enables the quadrupole isolation edges to be at m/z values that are theoretically impossible for doubly- and triply-charged tryptic peptide precursors. The precursor charge default state was set to two and the HCD collision energy was set to 27. The quadrupole isolation width was set to eight, and the MS2 resolution was 15,000. Halfway through the data acquisition of the individual samples, narrower isolation data was acquired on the pooled sample. This uses the same staggered isolation concept, except that the quadrupole isolation is set to m/z 4, and the targeted MS2 precursor values are staggered by m/z 2. The MS2 resolution is increased to 30,000, and the ion fill time is doubled to increase sensitivity and specificity for peptide detection. Instead of cycling through m/z 400 to 1000, these narrow isolation DIA runs are repeated six times where each injection covers a precursor range of m/z 100 (i.e., m/z 400–500, m/z 500–600, etc.). Hence, the full DIA data set was comprised of wide isolation runs for individual samples that covered the full precursor range of m/z 400 to 1000, plus a set of six narrow isolation runs acquired on a pooled sample. Wide isolation runs were also acquired for the pooled sample at the beginning, middle and end of each batch acquisition.

#### Data processing

Data analysis for the DIA data employed the computer program EncyclopeDIA v2.12.30 [25] to make a chromatogram library, and to then use that library to analyze the wide isolation DIA data for the individual samples. To make the chromatogram library, Prosit [26] was used to make an in silico spectral library for all tryptic peptides within a Uniprot human protein sequence FASTA file (Sept 2022). This Prosit-derived library was used to analyze the narrow isolation DIA data to obtain the chromatogram library used to analyze the wide isolation DIA data for the individual samples. Each of the three batches of samples had their own pool, which resulted in three chromatogram libraries. Only the 30,601 tryptic peptides detected in all three pools were considered moving forward. The results from EncyclopeDIA (an elib file containing chromatographic peak boundaries for each peptide) was used to import the wide isolation data into Skyline, which was used to remove questionable sample runs where the digestion and injection controls (yeast enolase and PRTC peptides) had anomalous intensities or retention times. Only two samples from the first batch had anomalous yeast enolase intensities (Figure S2), and those samples were excluded from all analyses. Peptides that had high variance (CV > 50%) within multiple pooled sample runs were also removed systematically from consideration. Based on the wide isolation data for the pooled samples in each batch, each of these peptides was normalized to the average signal intensity across the three batches (e.g., peptide X in batch 1 was normalized to the average signal of peptide X found in batches 1–3). Signal intensities for the few technical replicates were averaged first, and then any biological replicates were subsequently averaged, resulting in a csv file containing peptide rows and individual sample columns with cells containing the signal intensity (area under the chromatographic curve). Protein abbreviations are reported in results as their Uniprot IDs for consistency with the tables. Finally, peptides within each Uniprot ID were screened for large variations in their log2 Dementia/No Dementia values. If the log 2 ratios for one or more peptides differed by more than seven standard deviations from other peptides in the Uniprot IDs, these were separated into different protein groups for analysis. This analysis was used to separate, for example, peptides mapping to the Aβ sequence vs. those mapping to other regions of the amyloid precursor protein.

### Western blotting

To evaluate microvessel purity/enrichment, microvessels and capillary depleted brain homogenate obtained from a representative microvessel isolation were extracted in M-PER Mammalian protein extraction buffer (Thermo Fisher Scientific, cat no. 78501). 7.5ug of protein extract per well was separated on a Biorad Mini-Protean 4–20% TGX gel (cat no. 4561096) and blotted onto a nitrocellulose membrane using the iBlot 2 system using settings of 20 V for 1 min, 23 V for 4 min, and 25 V for 2 min. The membrane was blocked using Intercept blocking buffer (LICOR cat no. 927–60,001) for 1 h at room temperature and incubated overnight with antibodies against claudin-5 (Abcam cat no. ab15106, 1:500 dilution), MAP2 (EMD cat no. MAB3418, 1:1000 dilution), and beta actin (CST cat no. 8457S, 1:1000 dilution), diluted in blocking buffer. The secondary antibodies were IRDyes 680CW goat anti-mouse (LICOR cat no. 926–68,070) or 800CW goat anti-rabbit (LICOR cat no. 926–32,211). Blots were imaged using the Odyssey CLx (LICOR).

### Identification and analysis of cell-type enriched proteins:

We first utilized the ZEBRA database, an integrated single-cell gene expression atlas of the human and mouse brain, to identify enriched genes in different brain cell types of the neurovascular unit in human cerebral cortex. We specifically evaluated enriched genes in astrocytes, endothelial cells, mural cells, microglia, oligodendrocytes, fibroblasts, lymphocytes, and neurons. In screening for well-known markers of microvascular cell types such as claudin-5 (CLDN5), glial fibrillary acidic protein (GFAP), and aquaporin 4 (AQP4), we found that these genes appeared near or above a 3.5-Log2 fold level of enrichment, and thus set this as the threshold. Because we conducted this analysis manually, we further limited the number of enriched genes to the top 25 ranked by fold change from highest to lowest. We grouped subcategories of cell types (e.g., endothelial 1, endothelial 2) together to generate enriched gene lists. Non-protein coding genes or genes whose protein products were not found in the Uniprot database were excluded. These gene lists are shown in Table S10. We then determined which protein products of these enriched genes for each cell type were detected in our proteomics dataset, shown in Table S10. Notably, we detected no neuronal enriched proteins, whereas all other cell types of the NVU were represented in at least one enriched gene that was also detected at the peptide/protein level. All genes detected at the peptide/protein level were then cross-referenced against a second RNAseq database published by Yang et al. that evaluated cell-type specific gene expression changes in isolated brain microvessels from donors with or without Alzheimer’s disease using a method termed VINEseq [15]. We used this database to confirm findings of cell-type specific enrichment in ZEBRA by screening for increases in expression of at least 2 log2fold in the enriched cell type over most other cell types in the database. In many cases, gene expression was highest in the cell type of interest, but also detected at moderate levels (albeit with high variability) in a few other cell types; Table S10 recorded the order of expression from highest to lowest in each cell type and includes a determination of whether the gene exhibited cell-type specific expression or not. Gene/protein targets that did not show apparent cell-type specific enrichment in both databases were excluded from further analysis. Lymphocytes were excluded from further analysis because there was no apparent cell-type specificity for any of the enriched genes. The Yang VINEseq database was also used to further stratify positive hits, particularly for mural cells. Pericyte-specific proteins reported in ZEBRA were re-grouped into pericyte markers, smooth muscle cell markers, or general mural cell markers that label both smooth muscle cells and pericytes based on VINEseq subcategorization. Microglial genes were separated into microglia markers, perivascular macrophage markers, or common markers of both cell types based on VINEseq subcategorization. Table S10 includes the analysis done to generate the final list of cell-type specific proteins analyzed. The final list of cell-type specific genes detected at the protein level were evaluated for protein abundance in the AD and control groups and analyzed by two-way ANOVA and Sidak’s multiple comparisons test, comparing AD vs. No AD means for each protein.

### Statistical analysis

Significant differences in peptide signals were determined using the t-test after first performing a log2 transformation (the log2 transformed data was closer to a normal distribution). The p-values were then corrected for multiple hypotheses testing using the method of Benjamini–Hochberg with an alpha of 0.05. Differences in age distributions were compared using GraphPad Prism 8.4.3 using the Kolmogorov–Smirnov test. Two-way ANOVA with repeated measures was used to compute main effects and interactions of cell-type enriched protein changes, and Sidak’s multiple comparisons test compared means of each protein in AD vs. No AD groups. Data were analyzed using GraphPad Prism 8.4.3.

## Results

### Analysis of donor demographics

A unique feature of this study is the use of microvessels isolated from freshly collected postmortem brain tissue, as part of a rapid autopsy protocol, which allows for microvessel viability studies which we have previously described and characterized [20]. For this analysis, 28 females and 16 males met criteria for allocation into control (Overall ADNC score of 0–1, Braak stage ≤ IV, and no dementia) or AD (Overall ADNC score of 3, Braak stage V or VI, with dementia) groups with appropriate age matching within each sex (Table 1 and S1). All donors listed in Table 1 and Table S1 passed quality controls described in methods and were included in the analysis. The post- mortem interval was slightly but significantly longer for male control vs. male AD donors (Table 1); however, all post-mortem intervals were approximately 12 h or less and a difference of 2.5 h is considered to be negligible. We also noted that the age distribution of male donors (range: 58–93) significantly differed (p = 0.0006, Kolmogorov–Smirnov test) from that of the female donors (range: 78–99), with males being generally younger. One male with AD was younger than 65, meeting criteria for early-onset AD, but without a known genetic cause. We further evaluated age distributions of male and female donors over 55 years of age on repository at the UW BRaIN Lab and found that the male (n = 631) and female (n = 692) donor distributions significantly differed (p < 0.0001, Kolmogorov–Smirnov test) with males being younger. Thus, the generally lower age of males vs. females in this study cohort was expected based on repository demographics.

### Analysis of protein differences in AD

**Fig. 1:**
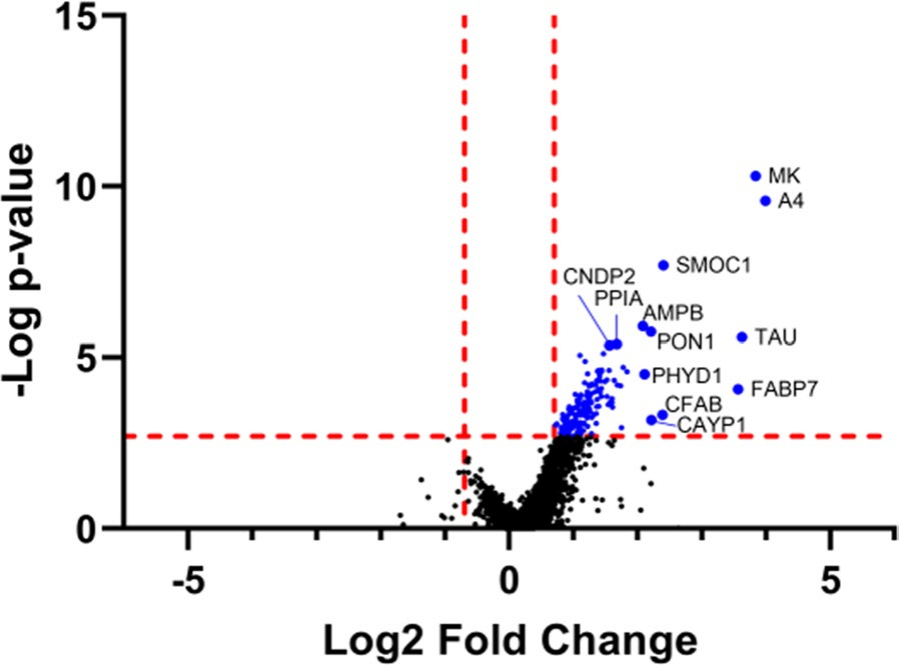
Volcano plot of protein changes in AD. Positive Log2 fold-changes indicate higher levels in AD vs. control, and negative Log2 fold-changes indicate lower levels in AD vs. control. Proteins that were significantly increased were identified by the Benjamini–Hochberg method using a 5% false discovery rate. P-value significance cutoffs for each group are indicated by the horizontal red dotted line. Vertical red dotted lines indicate the smallest fold-change among significantly increased proteins

**Fig. 2:**
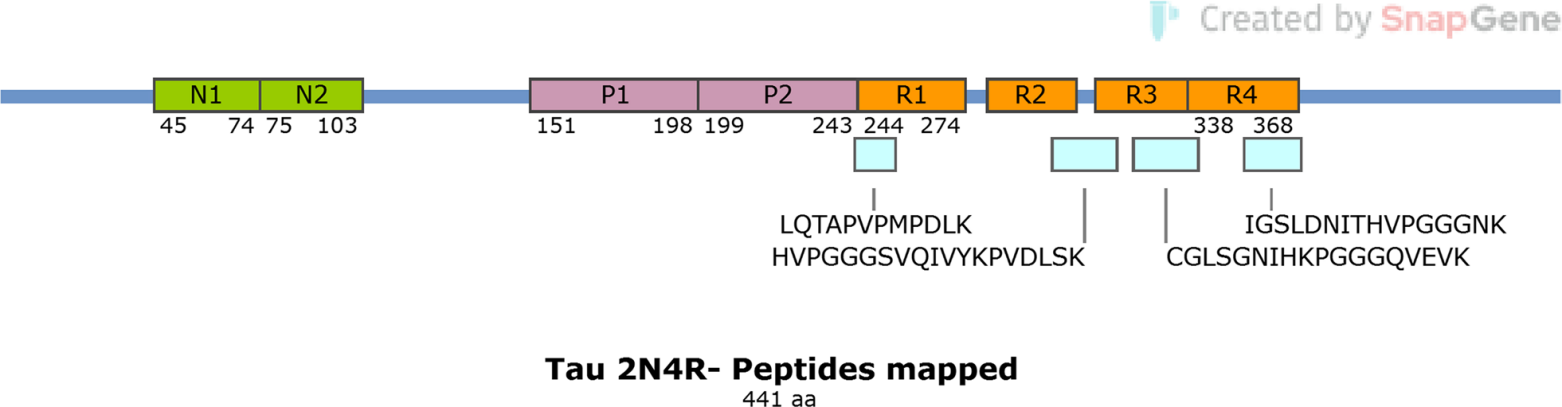
Mapping of tau peptides found to be increased in AD brain microvessels. Four peptides are mapped (one mapped peptide represents two nearly identical peptides that differed by a single lysine residue at the C-terminus, see Table S2) used to compute tau protein increases in AD. Peptides (blue) were mapped to the 2N4R (441aa) Tau protein sequence and coverage includes R1-R4 sequences. Figure 2 was prepared using SnapGene

Using approaches described previously to compute differences in protein levels between groups based on normalized peptide abundance [27, 28], we next determined which proteins were differentially expressed in AD vs. non-AD brain microvasculature. Male and female groups were analyzed separately, and as combined data to compute fold-changes in protein levels in the AD vs. control groups (Supplementary Tables 2–4). Peptide-level data are shown in Supplementary Tables 5–7. A summary list of significantly increased proteins, their abundance in AD vs. non-AD and abundance ratios, and p-values are shown in Supplementary Table 8. The volcano plot in Fig. 1 shows protein abundance changes in AD males and females combined. In the combined group, 168 proteins were significantly increased with AD, and none were significantly decreased. Among the increased proteins were A4 (just the two tryptic APP peptides mapping to the Aβ region) and Tau, the hallmark proteins comprising plaques and tangles of AD. A graphic of the aligned peptide sequences for Tau that were significantly increased in AD vessels are shown in Fig. 2 and indicates that a 4R form of Tau is enriched in human brain AD microvessels. Tau peptides that were detected but not significantly increased mapped to proline-rich regions, the R2 domain, and the C-terminus; no peptides of Tau’s N domains were detected. When we compared our list of significantly increased brain microvessel proteins to that of a recently published DIA proteomic analysis of inferior parietal lobe bulk tissue using the same methodology in subjects with or without AD [27], we found that A4, midkine (MK), SPARC related modular calcium binding 1 (SMOC1), neuroepithelial cell-transforming gene 1 protein (NET1), Glutathione S-transferase omega-1 (GSTO1), and platelet-activating factor acetylhydrolase IB subunit alpha1 (PA1B3) were common proteins that were found to be significantly increased in both datasets. The remaining 162 proteins were uniquely increased in brain microvessels from AD donors. When the male and female groups were analyzed separately, fewer proteins were found to be significantly increased in females (2 proteins: MK and A4) and males (3 proteins: A4, MK, and SMOC1).

**Fig. 3:**
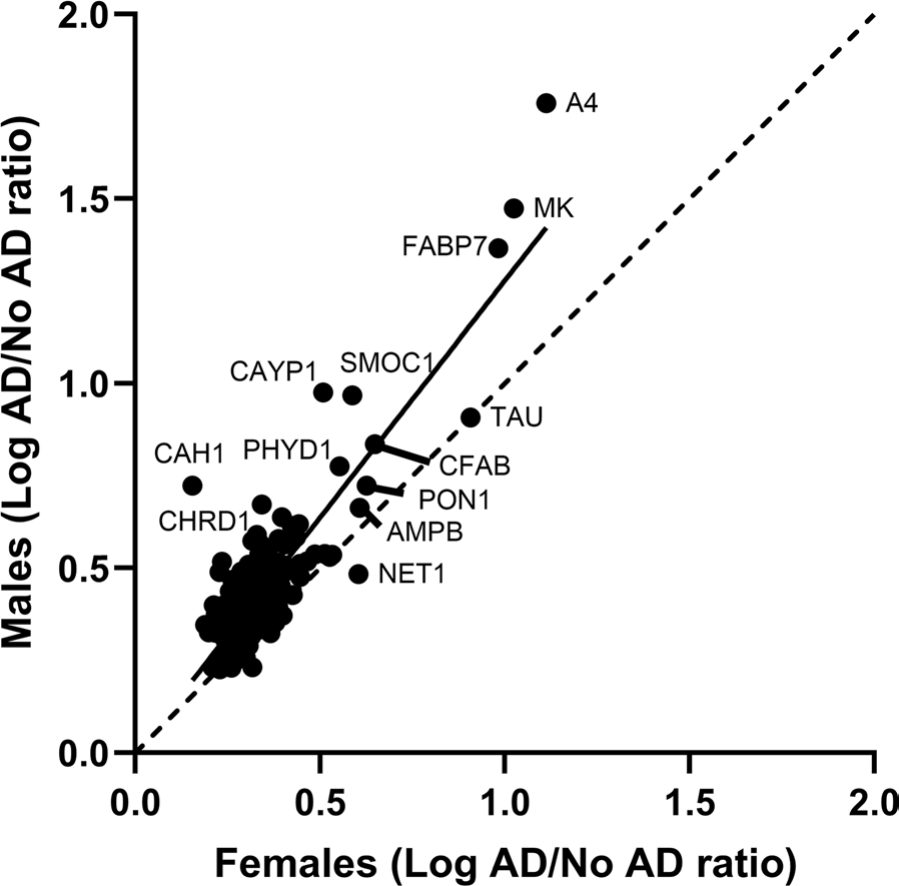
Linear regression analysis of the fold-changes of significantly increased proteins in AD brain microvessels, assessed in the male and female groups. The solid line is the best-fit line of the data. The dotted line is the line of identity (slope = 1)

We next evaluated the extent to which significant protein increases with AD that were found to occur in the combined group reflected trends in one or both sexes. Figure 3 shows a linear regression analysis of the log protein abundance fold-changes with AD in male and female groups for proteins that were increased in the combined group. The best fit line for this relation indicated that protein increases were larger overall in the male vs. female group. However, there was a strong positive correlation of protein changes in males vs. females (r-squared = 0.7346), verifying that the combined analysis represented protein changes happening in both male and female groups. We thus used the combined dataset in subsequent analyses.

**Fig. 4:**
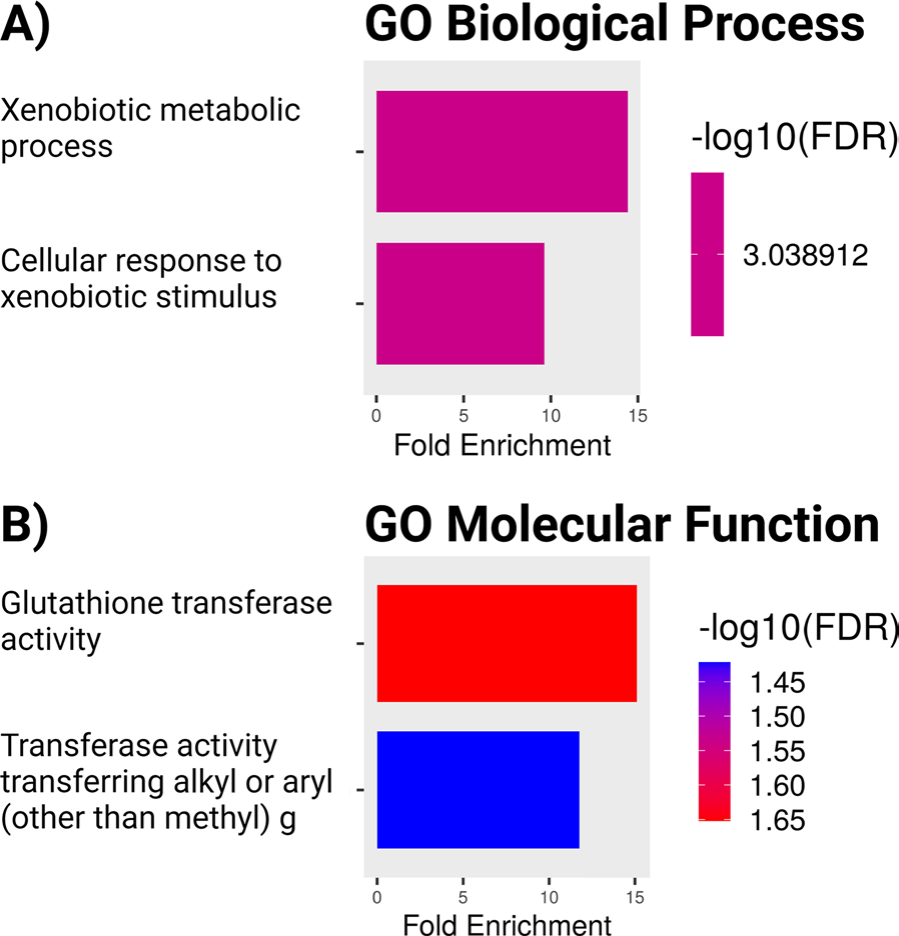
GO analysis of significantly increased proteins in AD. A) GO Biological Process, B) GO Molecular Function. The colors of the bars reflect the -log10(FDR), with ranges shown on the heat maps to the right of each graph. Figure 4 was prepared with the ShinyGO app and with BioRender.com

GO analysis of the 168 significantly increased proteins in AD was carried out using the ShinyGO v.0.80 app [29]. We evaluated the biological processes, cellular components, and metabolic functions pathway databases with FDR cutoff of 0.05 and limited analysis to the top 20 pathways meeting these criteria. The reference set was all proteins detected in the combined group. The analysis results are shown in Fig. 4 (Biological Process and Molecular Function) and Figure S3 (Cellular Component), and the proteins that grouped into each GO term are shown in Table S9. The most highly enriched GO pathways generally involved aspects of the xenobiotic response and glutathione transferase activity, reflected by the upregulation of the glutathione-S-transferases GSTP1, GSTM3, GSTO1, and GSTM2, carbonyl reductases CBR1 and CBR3, protein phosphatase 1F (PPM1F) which inhibits ferroptosis [30], and ubiquitin carboxyl-terminal hydrolase isozyme L1 (UCHL1), a deubiquitinase enzyme and known biomarker of brain injury [31]. The cellular component terms related to extracellular organelles, vesicles, and exosomes as well as extracellular space and regions. 34 proteins were common in all 5 terms. CBR3 and SMOC1 categorized with extracellular space and regions but not with the extracellular organelle, vesicle, and exosome terms. SMOC1 is a secreted extracellular matrix protein [32]. CBR3 is a cytosolic protein but is also designated as extracellular due to its detection in the human tear proteome [33]. Proteins with GO-terms of extracellular organelle, vesicle, or exosome were typically detected in various proteomic studies of extracellular vesicles in body fluids such as urine or plasma [34, 35], however most proteins also had intracellular locations and diverse functions according to Uniprot that were not clearly related to known aspects of extracellular vesicle formation or function [36]. We concluded from GO analysis that there was enrichment of pathways related to detoxification and the antioxidant response, particularly that of glutathione conjugation, but that the enrichment of cellular component pathways was largely driven by proteins identified in proteomic studies of exosomes in biofluids that also had important intracellular functions.

**Fig. 5:**
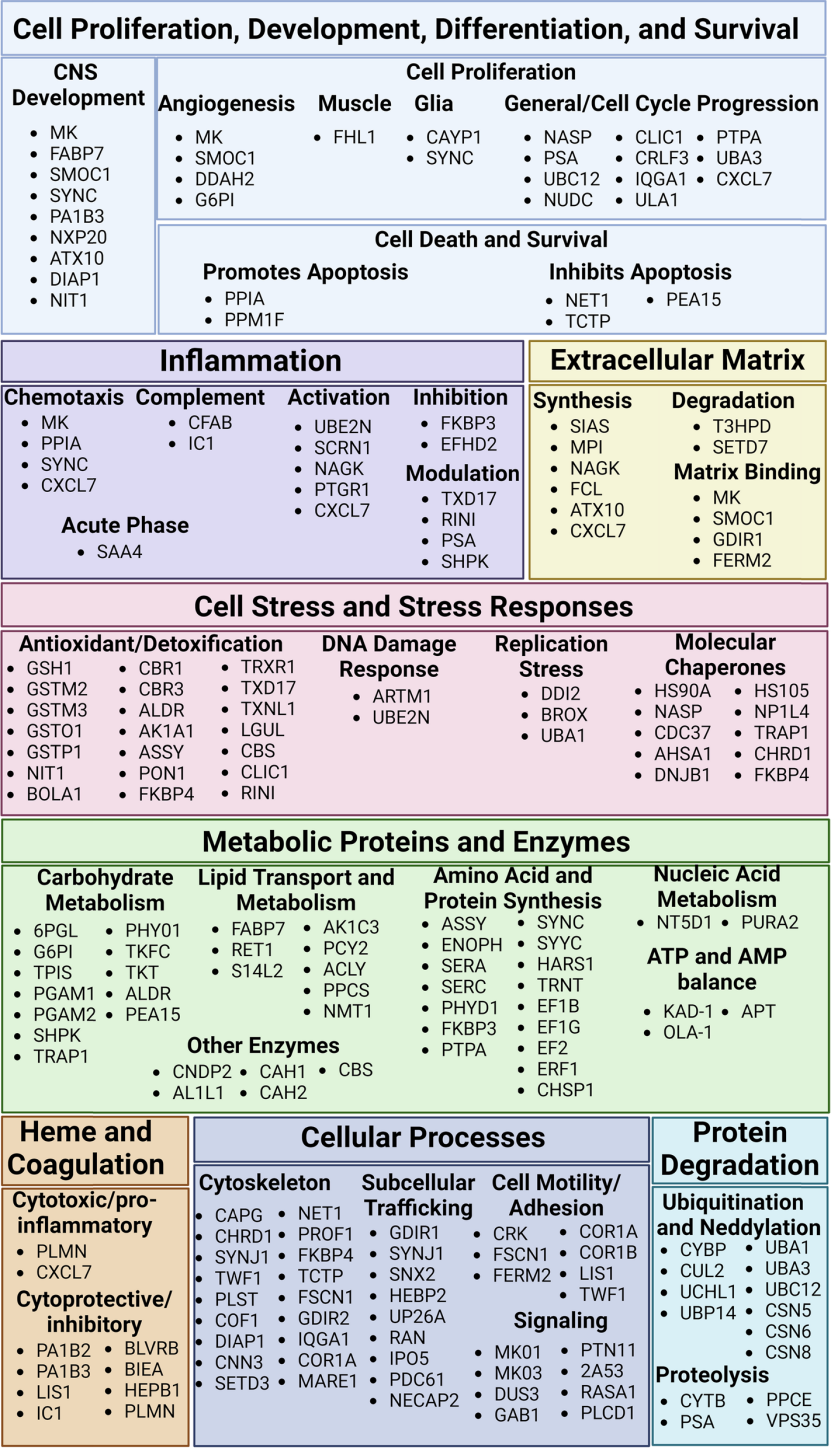
Functional categories analysis of the 168 significantly increased proteins in AD. Protein functions were systematically determined by checking the “Functions” section of the UniProt database, which includes references in support of protein functions. Common functional themes were then recorded, and proteins were categorized into groups and subgroups. Some proteins fell into multiple subgroups. Figure 5 was prepared with BioRender.com

To better understand the biological context of our findings, we systematically surveyed the list of 168 AD-increased proteins for their functions using the UniProt database. Using this approach, we identified functional themes and grouped proteins into functional categories within these themes. Some proteins fell into more than one category, and some proteins (CZIB, NHLC2, IPYR, and DP13A) either did not fall into any category or had unknown biological functions. The results are shown in Fig. 5. Overall, our functional themes analysis showed that increased brain microvessel proteins in AD included those that regulate cellular proliferation and brain development, apoptosis, inflammation, extracellular matrix, cellular stress responses, metabolism, coagulation responses, cytoskeletal changes, subcellular trafficking, and motility, cell signaling, and protein degradation.

**Fig. 6:**
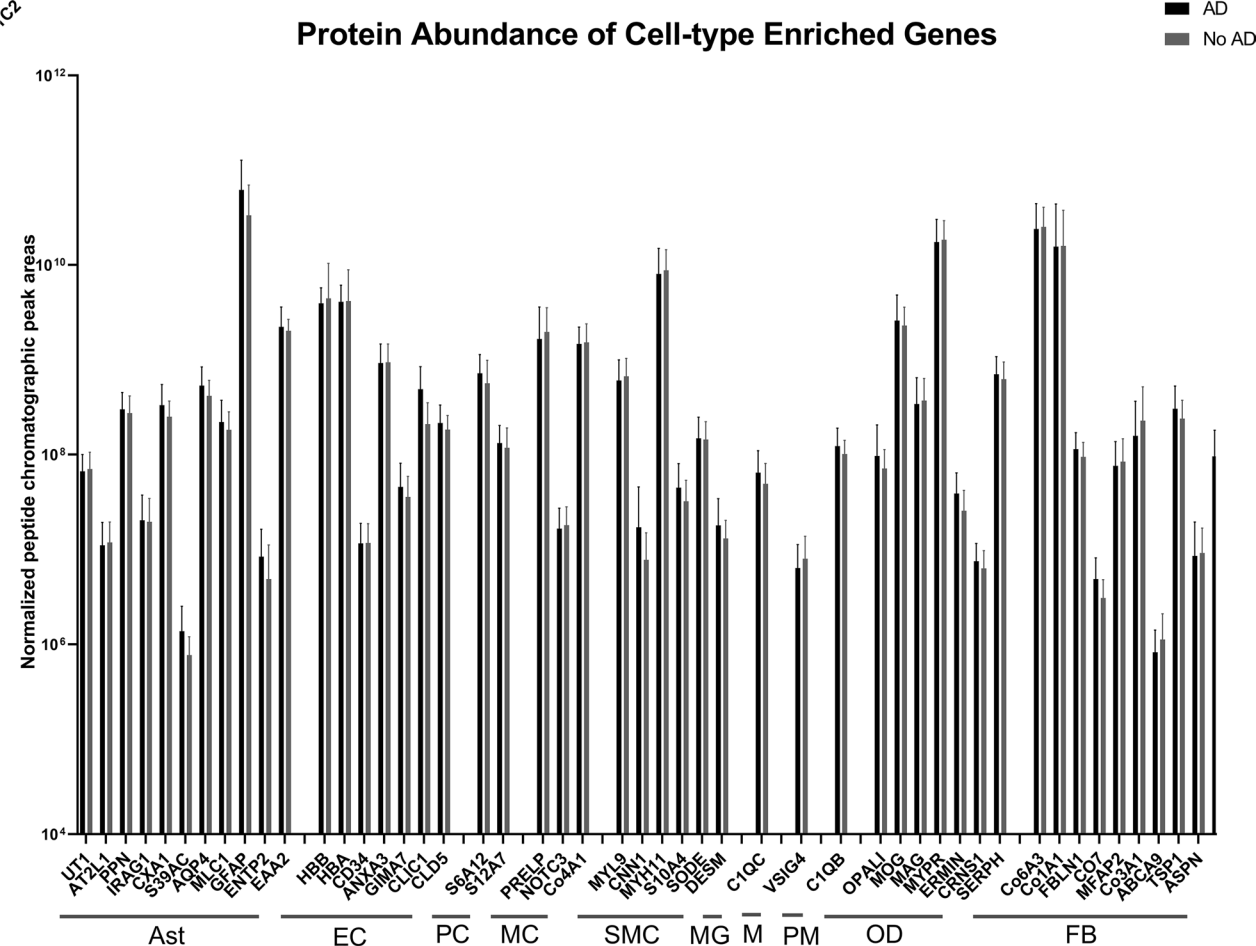
Cell-type enriched genes that were detected at the protein level in our dataset. Ast = astrocyte, EC = endothelial cell, PC = pericyte, MC = mural cell (genes enriched in both pericytes and smooth muscle cells), SMC = smooth muscle cell, MG = microglia, M = microglia/macrophage (genes found in both microglia and perivascular macrophages), PM = perivascular macrophage, OD = oligodendrocyte, FB = fibroblast

We next analyzed cell-type enriched genes of the brain microvasculature detected at the protein level in our dataset. The purpose of this analysis was to identify new and established cell-type specific protein markers of the brain microvasculature and details of the analysis are provided in Methods. The analysis to identify cell-type specific proteins is shown in Table S10. Notable findings from these analyses were that only a subset of cell-type enriched genes from the Zebra database were detected at the protein level for each cell type, and in some instances, there was discordance of apparent cell-type specificity in the two RNAseq datasets used for reference. No neuronally enriched genes were detected at the protein level, and lymphocytes were also not analyzed because we could not verify cell-type specificity of gene expression in the VINE-seq dataset. Figure 6 shows our analysis of cell-type specific proteins for abundance differences in AD vs. non-AD microvessels. Two-way ANOVA repeated measures analysis of proteins coded by our final list of cell-type enriched genes found no significant main effects of disease. There were significant main effects of subject (p = 0.0044, 2.054% of total variation), protein (p < 0.0001, 42.91% of total variation), and a significant protein x disease interaction (p < 0.0001, 2.711% of the total variation). A multiple comparison post-hoc test found no significant differences in means or statistical trends of individual proteins for the AD vs. control groups.

Finally, we assessed the extent to which significantly increased proteins in AD corresponded to gene expression changes. We analyzed all 168 proteins in our dataset for their corresponding gene expression changes in AD in the Yang single cell VINEseq database [15]. As statistical evaluations were limited by power and the number of multiple comparisons, we considered a given gene to be increased in a cell type if its averaged normalized counts at least doubled in AD, and if the error bars of the AD and control groups were not overlapping. Using this approach, we found that only 79/168 proteins appeared to have corresponding gene expression increases in at least one cell type. 9/168 proteins were not detected in any cell type at the mRNA level, and the remaining 80 were detected at the mRNA level but did not meet criteria for gene upregulation. Of the 79 genes that were upregulated in AD, 47 were upregulated in microglia, 11 in perivascular macrophages, 6 in T cells, 9 in meningeal fibroblasts, 3 in neurons, cortical astrocytes, and ependymal cells, 2 in arteriolar endothelial cells and arteriolar smooth muscle cells, and 1 in oligodendrocytes, oligodendrocyte precursor cells, transporter pericytes, and perivascular fibroblasts. 10 of the aforementioned genes were upregulated in more than one cell type. The proteins associated with AD-upregulated genes in each cell type are shown in Table S11.

## Discussion

Brain microvascular dysfunction is an important component of AD pathogenesis, but a more comprehensive understanding of the molecular changes occurring in the AD brain microvasculature is needed. RNAseq methods have elucidated meaningful changes of the brain microvascular transcriptome at the single-cell level [15], but a recent proteomic study of brain tissue highlights important protein changes that are not detected at the transcript level [16]. Two prior studies that have quantified changes in brain microvascular protein profiles in human control vs. AD tissues have been limited to sample sizes of 3–4 or 5–12 subjects per group [17, 18]. A larger, more recent study used a tandem-mass tag proteomics approach to compare proteomes of bulk brain tissue or brain microvasculature of the dorsolateral prefrontal cortex from subjects with AD, progressive supranuclear palsy (PSP), or controls [19]. In comparison, our study offers a comprehensive data set on peptide and protein abundance changes of the parietal cortex grey matter microvasculature in AD using a larger sample size of 21–23 subjects per group with low PMIs, age-matched within each sex, and with concordance of clinical and neuropathologic AD diagnosis. Proteomics was carried out using a novel, label-free, data-independent acquisition MS approach. Further, we isolated microvessels from freshly obtained brain tissue using protocols that have been optimized for downstream studies of viable human brain microvessels [20].

In our subject cohort, the ages of the male and female groups were matched within sex, with female age being higher than that of males. A generally younger age of male donors was also observed in the historical repository of donors, and thus highlights a population feature of donors in our region that may limit the ability to age-match between sexes for statistical comparisons when fresh tissue is collected over a limited sampling period. We found far fewer significant protein abundance changes in the male and female groups vs. the combined group, which we attribute to the smaller sample size of individual groups. Overall, we found that there was concordance of protein changes in males and females with AD, although the fold-increases in protein levels were generally higher in males than females. We speculate that this difference was due to the advanced age of the female group. We further note that it is common in studies of human AD to aggregate male and female demographic data such as age, so it is difficult to compare our cohort to others in the published literature. As female sex is a predominant risk factor for AD [37], analysis of both groups can be informative, even if the groups are not perfectly age matched.

Like our results, prior proteomic methods did not identify significant changes in cell-specific markers between AD and control groups [17–19], suggesting that the cellular composition and cell-type specific marker abundance of brain microvessels is not changing with AD. Although many brain cell type-specific markers were detected in our MV preps, we believe this is due to their known physical associations with the neurovascular unit rather than parenchymal contamination [5, 38]. This is supported in part by an absence of neuronal enriched proteins detected in our samples as well as our confirmation of MAP2 depletion in isolated brain microvessels shown in Figure S1. The VINEseq database by Yang et al. and the TMT proteomic analysis by Wojtas et al. which also used highly pure preps of human brain microvessels as starting material, reported mRNA expression or proteins from many brain cell types supporting their microvascular associations [15, 19]. However, 162/168 proteins in our label-free DIA proteomic analysis were uniquely increased in brain microvessels vs. bulk brain tissue [27], highlighting that there is a distinct microvascular pattern of protein changes in AD. This concept is also supported by findings of Wojtas et al. [19]. Our analysis also points to new cell-type specific markers of different human brain microvascular cell types verified to be present at the protein level and the relative abundance of their peptide sequences, which could be used to generate new antibodies for detection. For example, it was recently shown that SLC6A12 is a more ideal cell-type specific marker for human brain pericytes vs. conventional markers like PDGFRB [39]. Pericyte loss has been a noted feature of the AD brain microvasculature, however we did not observe apparent decreases in pericyte or mural cell-specific proteins. Further analysis is needed to evaluate aspects of pericyte dysfunction in isolated human AD brain MVs.

Our analysis of significantly increased proteins in the combined group identified Aβ and tau peptides as being among the most highly elevated AD brain microvascular proteins, which was expected since both proteins can deposit in the brain microvasculature as well as the parenchyma and contribute to brain microvascular dysfunction [40–43]. The tau peptides identified as being significantly increased mapped across all 4 microtubule-binding repeat regions of tau, supporting that an R4 form of tau accumulates in the AD brain microvasculature. We did not detect any tau peptides mapping to the N-terminal regions of tau, suggesting that tau fragments may be deposited in the brain microvasculature.

GO analysis identified the significant enrichment of xenobiotic response pathways and glutathione transferase activity. In the functional themes analysis, we noted increased abundance of proteins involved in glutathione synthesis and conjugation. These included glutamate-cysteine ligase catalytic subunit (GSH1), which catalyzes the first and rate-limiting step of glutathione synthesis [44], as well as glutathione s-transferases GSTM2 and 3, GSTO1, and GSTP1 that conjugate glutathione to a variety of compounds and are critical for detoxification of endogenous and exogenous substances and neutralization of toxic lipid oxidation products [45]. Also increased was deaminated glutathione amidase (NIT1) which catalyzes a metabolite repair reaction to dispose of the harmful deaminated glutathione [46]. GSTO1 was also increased in bulk brain tissue [27], indicating that glutathione-S-transferase increases are not limited to the brain microvasculature. The VINE-seq [15] database indicated that expression of respective glutathione-related genes was abundant across most cell types, and did not appear to increase for any of these proteins in AD. Additional proteins identified to be involved in cellular detoxification included proteins that reduce carbonyls (carbonyl reductases CBR1 and CBR3, Aldo–keto reductases ALDR and AK1A1). Carbonyls are products of oxidative damage to lipids, proteins, and carbohydrates that are increased in AD and contribute to AD pathogenesis [47]. Another notable protein with antioxidant functions included Paraoxanase-1 (PON1) which is a lipoprotein enriched on high-density lipoproteins (HDL) with anti-atherosclerotic functions. PON1 has also been implicated in AD, although its precise functions are complex [48]. Although we could not rule out the possibility that the enrichment of some proteins implicated in xenobiotic detoxification was related to pharmacotherapies for AD, we found no evidence in the literature to support this possibility. Additionally, most older adults with or without AD have high levels of prescription and over-the-counter drug use [49, 50], high levels of medication non-adherence [51], and those with advanced AD often discontinue their AD medications due to increasing risk relative to benefit [52]. Thus, we interpret the increases in proteins with antioxidant and detoxification functions in AD as likely to be cytoprotective responses to AD-related oxidative stress and toxic metabolite production.

Additional functional categories of proteins included those known to be upregulated in brain development and those that regulate cellular proliferation as well as cell death and survival. Remarkable proteins in this category include MK, a protein that is upregulated in the brain during mid-gestation and that promotes angiogenesis during human brain development through interactions with endothelial and mural cells [53, 54]. In the adult, midkine mRNA levels remain low in the brain throughout the lifespan [53], although midkine expression is increased in the brain following injury and appears to be neuroprotective in the immediate stages [55] but may be harmful for longer-term outcomes [56]. Bulk brain proteomic analyses identified discordance of MK mRNA and protein levels in AD [16, 27]. SMOC1 is another protein that promotes angiogenesis during embryonic brain development- it is secreted by differentiating neurons and promotes the proliferation of endothelial cells by activating the transforming growth factor beta receptor 1 and phospho-2/3 SMAD signaling [57]. SMOC1 levels were also shown to be increased by hypoxia in cultured endothelial cells [58]. MK and SMOC1 are also noted for their interactions with the extracellular matrix [59, 60]. Additional proteins involved in cell cycle progression were increased as well, suggesting a proliferative state of brain microvascular cells in AD. It has been proposed that brain angiogenesis is a pathological feature of AD, which could be triggered by changes in cerebral blood flow, responses to inflammatory insults, accumulation of Aβ, and other features of AD [61]. We detected many proteins with inflammatory functions that were increased in abundance, although most of these have underexplored functions in AD brain microvasculature. Microglia can also proliferate in response to AD pathology [62]. The apparent increase in proliferation of brain microvascular cells may explain, in part, why proteins involved in metabolic processes such as glycolysis and protein synthesis are increased, as are proteins involved in the response to replication stress and DNA damage. Another proteomic analysis by Suzuki et al. showed that ribosomal proteins and proteins involved in glycation were increased in AD brain microvessels from neocortical grey and white matter [17]. We did not identify upregulation of the same ribosomal proteins in our analysis, possibly due to methodological differences, donor differences, or other features of study design. However, we did observe increases of many proteins involved in protein translation such as tRNA ligases (SYNC, HARS1) and translation regulatory factors (ERF1, EF1B). We posit that higher levels of stress-related protein synthesis and cellular proliferation could partly explain why we only observed significant increases in protein abundance, and no significant decreases in AD brain microvessels. A recent proteomic analysis of brain microvascular protein changes in an infection-induced vasculopathy model also found that significantly altered proteins were greater in abundance [63], further supporting that protein increases could reflect a global stress response of the brain microvasculature.

Among proteins that were significantly increased in abundance, we noted that some can control BBB functions. Peptidyl-prolyl cis–trans isomerase A (PPIA), otherwise known as cyclophilin A, was previously shown to be upregulated in pericytes and to promote BBB leakage via inflammatory and matrix metalloproteinase (MMP) activation, particularly in humanized *APOE4* mice [64]. MMPs can contribute to BBB leakage by degrading components of the extracellular matrix and tight junctions [65]. We did not, however, observe decreases in tight junction proteins: for example, AD/No AD ratios for claudin 5 and occludin were 1.18 and 1.38, respectively (Table S2). We detected increases in plasminogen and proteins involved in coagulation and heme breakdown, suggesting that vascular leakage to blood components and/or vascular coagulation processes were higher in brain microvessels of AD vs. no AD subjects. We also noted that BBB-protective processes were activated. For example, Netrin-1 (NET-1) was increased in our dataset and has been shown to promote BBB integrity under inflammatory conditions by upregulating tight junction proteins [66]. Overall, these findings indicate that both damage-inducing and damage repair processes are increased in AD brain microvessels. We also investigated the dementia/no dementia fold-changes in key BBB transporters of Aβ, such as the low-density lipoprotein receptor-related protein-1 (LRP-1, Uniprot ID LRP1) [67–69], P-glycoprotein (P-gp, Uniprot ID MDR1) [70, 71], and receptor for advanced glycation end products (Uniprot ID RAGE) [72], and the transporter of glucose (GLUT1, Uniprot ID GTR1) [73, 74]. All of these transporters except RAGE were detected in our dataset (Table S2), however LRP-1, P-gp, and GLUT1 had ratios of 1.0327, 1.0553, and 1.027, respectively, indicating that there was no apparent change in protein abundance. Prior studies corroborate discordance of transporter protein abundance and transporter function, particularly for P-gp in AD [75] and for LRP-1 under inflammatory conditions [76]. Although decreased cerebrovascular protein levels in AD have been reported for LRP-1, P-gp, and GLUT-1 previously [67, 73, 77], we posit that the apparent discordance of our results reflects the heterogeneity of AD subject populations. Tissue availability limits mechanistic validation studies in this cohort, but these studies are ongoing in additional samples with similar clinical and neuropathologic criteria.

Like proteomic findings of bulk brain tissue from AD subjects [16], we found that many of the proteins that were increased in AD brain microvessels did not show corresponding increases in gene expression. Most of the concordant gene expression increases were exclusively in microglia, highlighting their importance in AD pathophysiology, and possible molecular links of microglia-specific interactions with the brain vasculature. The apparent specificity for microglia is not clearly due to higher numbers of microglia associating with the vasculature, as microglia-specific markers were not increased with AD at the protein level. Instead, we posit that the changes reflect altered transcriptional profiles of vascular-associated microglia in AD. A limitation of our analysis is that it is unknown whether the brain microvascular proteins detected arise from cells that are physically associated with the microvasculature, or whether they are released into interstitial fluid and are subsequently bound or internalized by other microvascular cell types. Further assessment is needed to understand the microglia-vascular interactions that may occur in AD and drive AD pathogenesis. A limitation of this analysis comparing RNA to protein levels is that two different cohorts of subjects were compared; future studies that evaluate both mRNA and protein profiles from the same donor would offer stronger support on the congruence of mRNA and protein levels (or lack thereof) in AD. Overall, however, these data suggest that both transcriptional and post-transcriptional mechanisms such as increased protein translation, vascular protein binding, protein uptake and retention, impaired degradation, or others are contributing to protein increases in the AD microvasculature.

## Conclusions:

In summary, our proteomic analysis offers a comprehensive reference for evaluating brain microvascular protein changes in AD. Our sub analysis also highlights the utility and value of existing publicly available datasets that can be used to inform proteomic findings and identify targets that consistently change across studies from different labs/in different subject populations. The protein profiles identified here suggest that AD brain microvessels have an overall pattern of increased protein levels vs. controls, which may be due to transcriptional or post-transcriptional mechanisms that are part of an adaptive response to compensate for increased metabolic demands or replication stress. Our results support the biological conclusion that AD microvessels exist in a stressed state, but they also appear to compensate, particularly for oxidative stress, by increasing proteins that mediate cellular detoxification and oxidant neutralization. Associated with this stressed state is the increase of proteins that are known inducers of BBB disruption, as well as those whose upregulation protects key BBB functions. Overall, our proteomic findings provide a holistic view of the brain microvascular stress response landscape that is likely in place to preserve key microvascular functions in AD. Importantly, cytoprotective responses to AD are understudied in general, and so our findings highlight a novel avenue of AD brain microvascular pathophysiology. Future mechanistic studies in viable brain microvessels are ongoing and an important direction for this work.

## Supplementary Information

Supplementary Figure 1. Western blot demonstrating enrichment of isolated brain microvessels. L= ladder, with numbers on the left indicating the molecular weights of the ladder markers in kDa, P= parenchymal fraction that was capillary-depleted, MV = microvessel fraction. Figure S1 was prepared with BioRender.com.

Supplementary Figure 2. The median-normalized chromatographic peak areas for each sample are shown for one peptide from the digestion control, enolase (top panel) and for the LCMS injection control (bottom panel) in the first batched run. Yeast enolase protein was added to each sample in the batch prior to tryptic digestion. The Pierce Retention Time Control (PRTC) peptide mixture was added after digestion and prior to injection to the LCMS. The digestion controls for samples 14_7 and 28_12 (indicated by arrows) were anomalously low even though the LCMS injection control had peak areas similar to the other samples. We assume that the tryptic digestion for those two samples had failed or was somehow significantly different from the rest. Hence, those two samples were excluded from the subsequent data analysis. No samples from subsequent batched runs required exclusion.

Supplementary Figure 3. GO analysis of Cellular Component enriched pathways among significantly increased proteins in AD. Significant pathways identified in this category appeared to be driven by detection of these proteins in exosomes from human tears and other biofluids in studies unrelated to AD. The colors of the bars reflect the -log10(FDR), with ranges shown on the heat maps to the right of each graph. Figure S3 was prepared using the ShinyGO app and with BioRender.com.

Supplementary Figure 4.

Supplementary Table 1.

Supplementary Tables 2-4.

Supplementary Tables 5-7.

Supplementary Table 8.

Supplementary Table 9.

Supplementary Table 10.

Supplementary Table 11.

## Data Availability

No datasets were generated or analysed during the current study.

## Abbreviations

ACT: Adult Changes in Thought
AD: Alzheimer’s disease
ADNC: Alzheimer's disease neuropathologic change
ADRC: Alzheimer's disease research center
AQP4: Aquaporin 4
Aβ: Amyloid beta
BBB: Blood–brain barrier
CLDN5: Claudin 5
CLIC1: Chloride intracellular channel 1
DIA: Data-independent acquisition
DPBS: Dulbecco's Phosphate Buffered Saline
EC: Endothelial cell
FABP7: Fatty acid binding protein 7
GFAP: Glial fibrillary acidic protein
GSH1: Glutamate-cysteine ligase catalytic subunit
GSTO1: Glutathione-S-transferase omega-1
HDL: High-density lipoprotein
IRB: Institutional Review Board
KPWHRI: Kaiser Permanente Washington Health Research institute
LC/MS: Liquid chromatography/Mass spectrometry
MK: Midkine
MMP: Matrix metalloproteinase
NET-1: Netrin-1
NIT1: Deaminated glutathione amidase
PAIB3: Platelet-activating factor acetylhydrolase 1B subunit alpha 1
PIPA: Peptidyl-prolyl cis–trans isomerase A/cyclophilin A
PMI: Post-mortem interval
PON1: Paraoxanase-1
SMOC1: SPARC related modular calcium binding 1
UW: University of Washington
VWF: Von Willebrand Factor
