# Supplementary figures and images for: Data-independent acquisition proteomic analysis of the brain microvasculature in Alzheimer’s disease identifies major pathways of dysfunction and upregulation of cytoprotective responses
Source: Fluids Barriers CNS. 2024 Oct 21;21:84. doi: 10.1186/s12987-024-00581-1 (PMC11492478; doi:10.1186/s12987-024-00581-1)

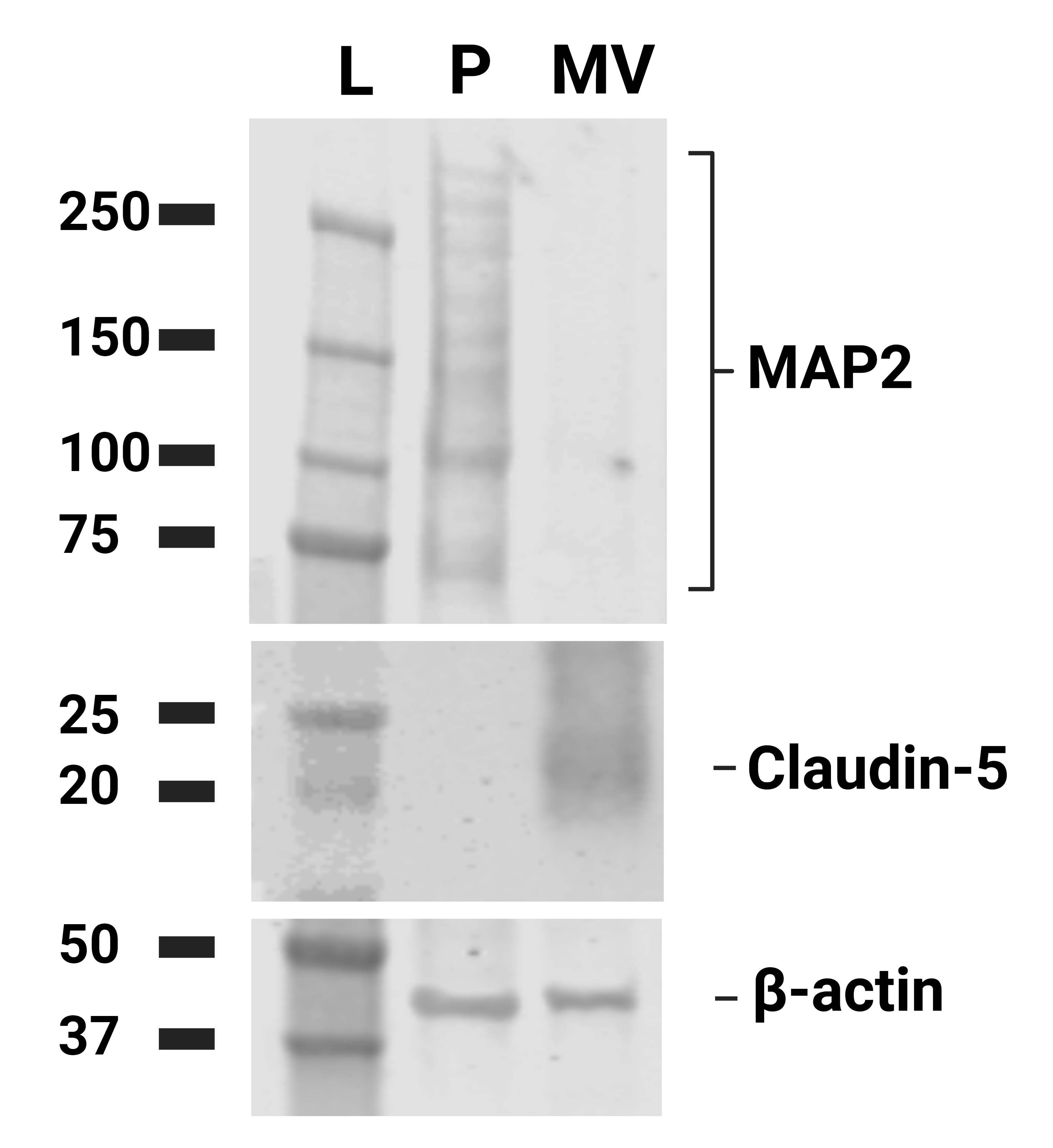

Supplement: Supplementary file 1 — Supplementary Figure 1. Western blot demonstrating enrichment of isolated brain microvessels. L= ladder, with numbers on the left indicating the molecular weights of the ladder markers in kDa, P= parenchymal fraction that was capillary-depleted, MV = microvessel fraction. Figure S1 was prepared with BioRender.com. [file 12987_2024_581_MOESM1_ESM.png]

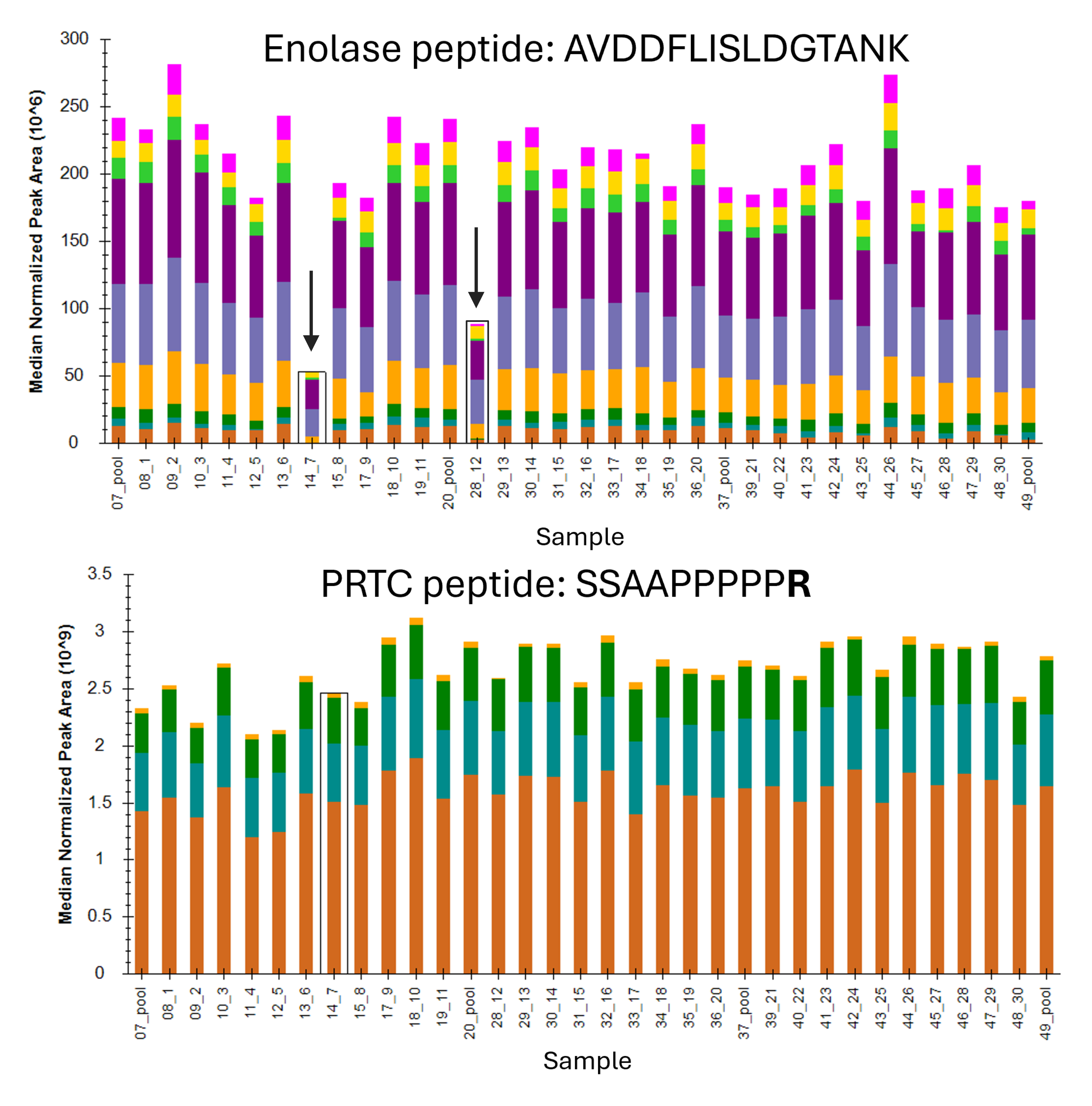

Supplement: Supplementary file 2 — Supplementary Figure 2. The median-normalized chromatographic peak areas for each sample are shown for one peptide from the digestion control, enolase (top panel) and for the LCMS injection control (bottom panel) in the first batched run. Yeast enolase protein was added to each sample in the batch prior to tryptic digestion. The Pierce Retention Time Control (PRTC) peptide mixture was added after digestion and prior to injection to the LCMS. The digestion controls for samples 14_7 and 28_12 (indicated by arrows) were anomalously low even though the LCMS injection control had peak areas similar to the other samples. We assume that the tryptic digestion for those two samples had failed or was somehow significantly different from the rest. Hence, those two samples were excluded from the subsequent data analysis. No samples from subsequent batched runs required exclusion. [file 12987_2024_581_MOESM2_ESM.png]

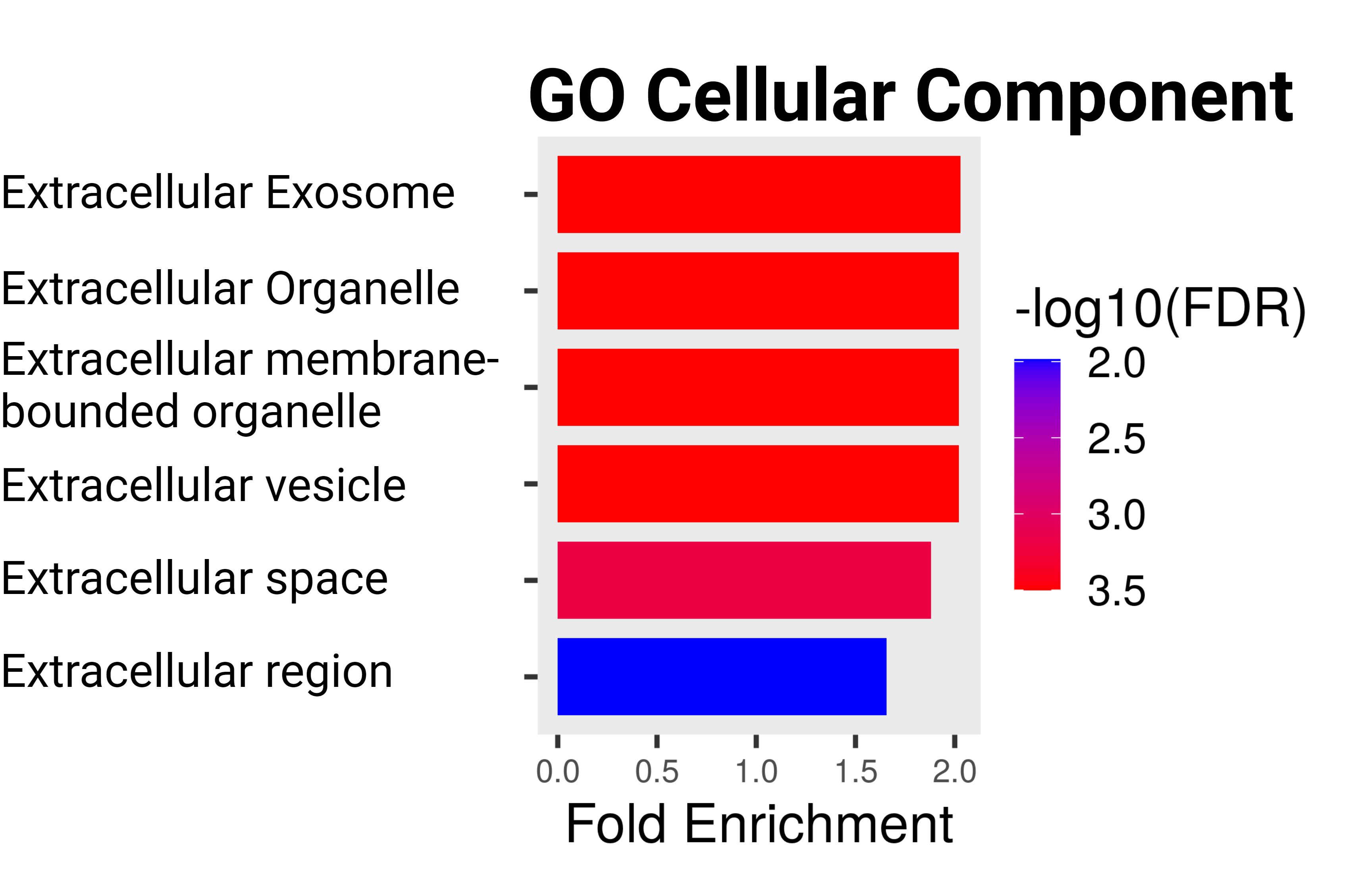

Supplement: Supplementary file 3 — Supplementary Figure 3. GO analysis of Cellular Component enriched pathways among significantly increased proteins in AD. Significant pathways identified in this category appeared to be driven by detection of these proteins in exosomes from human tears and other biofluids in studies unrelated to AD. The colors of the bars reflect the -log10(FDR), with ranges shown on the heat maps to the right of each graph. Figure S3 was prepared using the ShinyGO app and with BioRender.com. [file 12987_2024_581_MOESM3_ESM.png]
