# Supplementary material for: Data-independent acquisition proteomic analysis of the brain microvasculature in Alzheimer’s disease identifies major pathways of dysfunction and upregulation of cytoprotective responses
Source: Fluids Barriers CNS. 2024 Oct 21;21:84. doi: 10.1186/s12987-024-00581-1 (PMC11492478; doi:10.1186/s12987-024-00581-1)
Supplement: Supplementary file 4 — Supplementary Figure 4. [file 12987_2024_581_MOESM4_ESM.pdf]

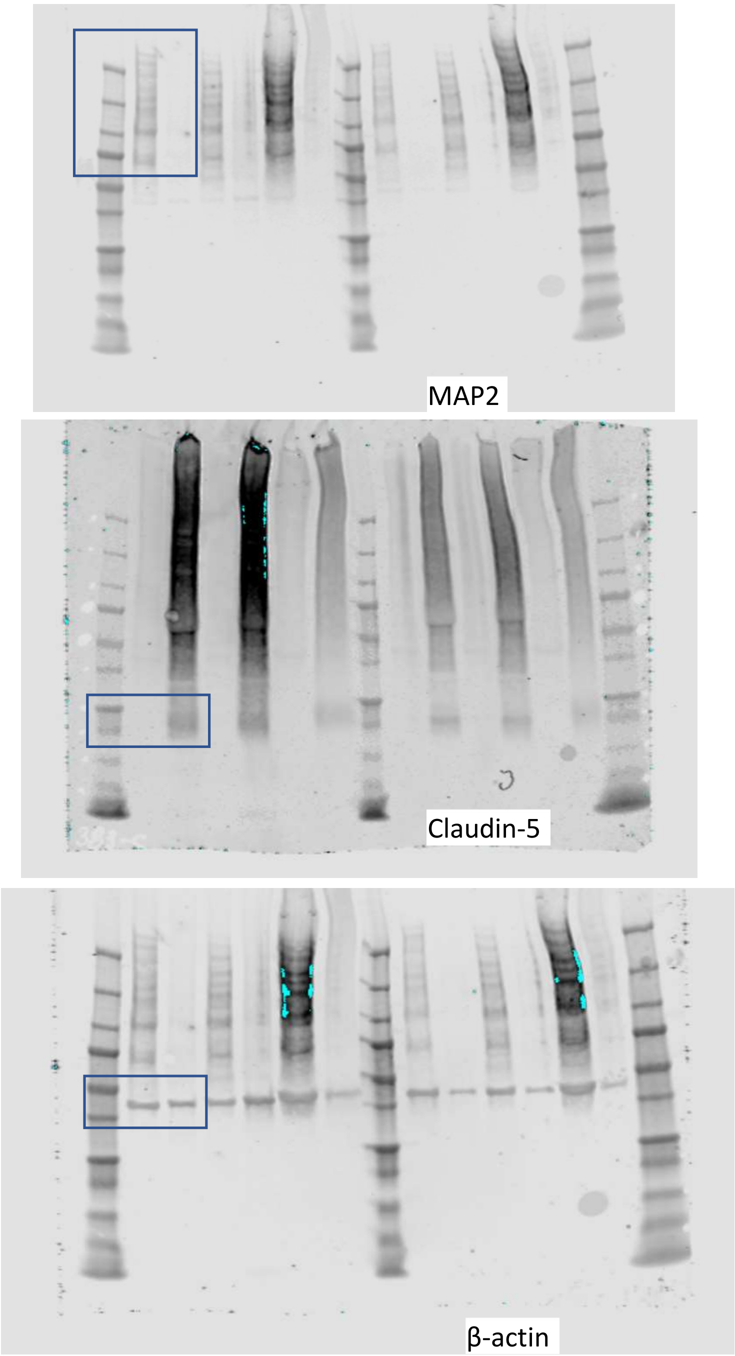

Figure S4: Whole Uncropped blots of MAP2, Claudin-5, and β-actin used to generate Figure S1. Blue boxes indicate the bands used to make Figure 1.
